# Hypofractionated Radiotherapy in Head and Neck Cancer Elderly Patients: A Feasibility and Safety Systematic Review for the Clinician

**DOI:** 10.3389/fonc.2021.761393

**Published:** 2021-11-12

**Authors:** Antonio Piras, Luca Boldrini, Sebastiano Menna, Valeria Venuti, Gianfranco Pernice, Ciro Franzese, Tommaso Angileri, Antonino Daidone

**Affiliations:** ^1^ UO Radioterapia Oncologica, Villa Santa Teresa, Palermo, Italy; ^2^ Dipartimento di Diagnostica per Immagini, Radioterapia Oncologica ed Ematologia, UOC Radioterapia Oncologica—Fondazione Policlinico Universitario Agostino Gemelli (IRCCS), Rome, Italy; ^3^ Radioterapia Oncologica, Università degli Studi di Palermo, Palermo, Italy; ^4^ UO Oncologia, Fondazione Istituto G. Giglio, Palermo, Italy; ^5^ Radiotherapy Department, Humanitas Clinical and Research Hospital Istituto di Ricovero e Cura a Carattere Scientifico (IRCCS), Milan, Italy; ^6^ Biomedical Science Department, Humanitas University, Milan, Italy; ^7^ UO Radiologia, Villa Santa Teresa, Palermo, Italy

**Keywords:** radiotherapy, elderly, hypofractionation, geriatric oncology, elderly oncology, head and neck cancer

## Abstract

**Objective:**

Radiotherapy (RT) in the head and neck (H&N) site are undoubtedly the most challenging treatments for patients. Older and frail patients are not always able to tolerate it, and there are still no clear guidelines on the type of treatments to be preferred for them. The recommendations for Risk-Adapted H&N Cancer Radiation Therapy during the coronavirus disease 2019 (COVID-19) pandemic provided by the ASTRO-ESTRO consensus statement achieved a strong agreement about hypofractionated RT (HFRT). A systematic literature review was conducted in order to evaluate the feasibility and safety of HFRT for older patients affected by H&N malignancies.

**Materials and Methods:**

A systematic database search was performed on PubMed and Embase according to Preferred Reporting Items for Systematic Reviews and Meta‐analyses (PRISMA) guidelines. Original studies, case series, and case reports describing the use of HFRT (with at least 2.2 Gy fractions) in patients with mean age ≥65 years were included. The analysis was based on the type of study, number of patients, mean age, tumor site, histology, performance status (PS), RT details, concomitant chemotherapy (CT), and described clinical outcomes. All the reported doses have been calculated in equivalent dose in 2 Gy fractions (EQD2) and biologically effective dose (BED) using α/β = 10 Gy or α/β = 12 Gy.

**Results:**

We selected 17 papers that met the inclusion criteria and divided them in 4 categories: 6 articles analyze HFRT performed twice daily in repeated cycles, 3 once a day in repeated cycles, 4 in alternative days, and the last 4 in consecutive days.

**Conclusion:**

HFRT seems to be a good treatment with an acceptable prolonged disease control. In older patients fit for radical treatments, a 55 Gy in 20 fractions regimen can be proposed as a valid alternative to the standard fractionated RT, but there are a multitude of hypofractionated regimens, ranging from single fraction, quad shot, and 1-, 2-, 3-, 4-, and 5-week schedules that all may be appropriate. The correct regimen for a patient depends on many factors, and it represents the result of a more specific and complex decision.

## Introduction

The average age of the world’s population is increasing, and an absolute number of cancer patients aged 65 years or more is expected to double in the next 20 years worldwide (1, 2).

As these patients are often frail and unfit for multimodal oncological therapies and need specific management, increasing importance is being acknowledged to the figure of the oncogeriatrician, with the aim of identifying elderly patients who able to undergo active cancer treatments, among which radiotherapy plays a relevant role both with curative or palliative care intent (3–5).

Considering the common logistic difficulties that frail patients suffer during the potentially prolonged and complex multimodal cancer therapies, there is a tendency to perform hypofractionated radiotherapy (HFRT) treatments, aiming to achieve early clinical results (i.e., symptoms reduction) and reduce patients’ and caregivers discomfort of having to reach the hospital for several days (6–9).

Radiation treatments in the head and neck (H&N) site are undoubtedly the most challenging treatments for patients, both for their overall duration and for the common toxicities that may lead to severe mucositis, dysphagia, and consequent malnutrition and general impairment (10–12).

Elderly and frail patients are not always able to tolerate such treatments and are therefore often referred exclusively to palliative treatments (13). Despite the significant clinical and societal burden of such cases, there are still no clear guidelines on the type of treatments to be preferred in this specific patient setting.

The severe acute respiratory syndrome coronavirus 2 (SARS-CoV-2) outbreak has renewed the interest in HFRT, in general, and for locally advanced H&N cancer, in particular, thanks to the reduction in the risk of getting exposed to the virus by reducing the number of visits to the hospital and the interaction with healthcare professionals (14, 15).

The recommendations for Risk-Adapted H&N Cancer Radiation Therapy during the COVID-19 pandemic provided by the ASTRO-ESTRO consensus statement achieved a strong agreement about HFRT (16).

Furthermore, besides the evident logistical advantages, radiobiological modeling suggests that 3.0 Gy per fraction or accelerated hyperfractionation schedules (1.8 Gy per fraction with two fractions per weekday) are considerably more effective for H&N tumor control and for the reduction in late effects than the standard 2.0 Gy fractionation (17).

Finding an HFRT schedule with radical intent or with the aim of a prolonged local control (LC), which can be reached without too much discomfort and toxicity, would be significantly advantageous in this group of patients.

A systematic literature review was performed in order to evaluate the feasibility and safety of HFRT for elderly patients affected by H&N malignancies.

## Materials and Methods

A systematic database search was conducted using definite keywords, according to Preferred Reporting Items for Systematic Reviews and Meta‐analyses (PRISMA) guidelines (18).

The search strategy was performed on PubMed and Embase using the search terms: “hypofractionated radiotherapy head and neck cancer elderly,” “head and neck cancer hypofractionated radiotherapy,” and “head and neck cancer hypofractionated radiation therapy.”

Original studies, case series, and case reports describing the use of HFRT (with at least 2.2 Gy fractions) in patients with mean age ≥65 years were included in this review.

Papers about re-irradiation, not focused about H&N or elderly patients, were excluded from the analysis. In addition, studies performed on animals or not in English were also discarded.

The analysis was based on the type of study, number of patients, mean age, tumor site, histology, performance status (PS), RT details, concomitant chemotherapy (CT), and reported clinical outcomes.

All the doses have been calculated in equivalent dose in 2 Gy fractions (EQD2) and biologically effective dose (BED) using α/β = 10 Gy or α/β = 12 Gy (19).

## Results

A total of 766 papers were found at the first search, 456 of which were duplicates. Of the 310 remaining results, 253 were excluded after a careful screening of abstracts. Fifty-seven articles were lastly selected for further accurate analysis. Out of these, 40 were excluded, as they did not directly target H&N cancer, elderly patients, or HFRT. The remaining 17 papers were lastly included in this review (20–35).

**Figure 1:**
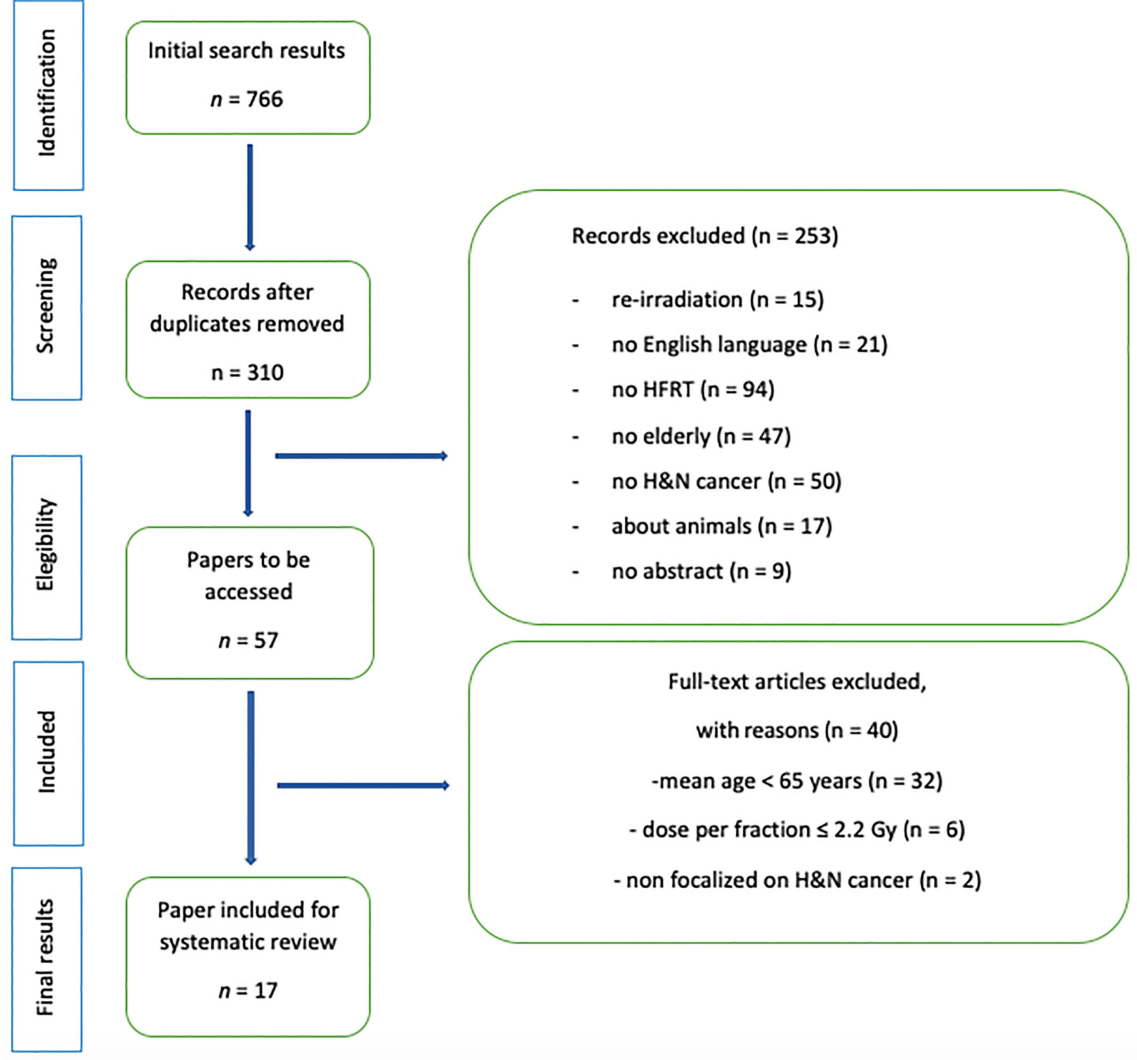
PRISMA diagram.

All the review workflow was compliant with the PRISMA guidelines, and the relative flowchart is reported in **Figure 1** (18).

We reported the characteristics of the reviewed studies in **Table 1**.

**Table 1:** Studies and patients characteristics.

First author, year	Type of study	No. of pts	Mean age (range)	Tumor site	Histotype	PS before treatment	CT	Outcome
Ferro (20)	Prospective	17	85 (80–97)	23.5% larynx	64.7% SCC	ECOG:	No	PS improved in 76.5%; stable in 23.5%; No G3 toxicity; SFS 83.3% in 9 pts; 87.5% in 8 pts; OR 88% (29% CR, 59% PR, 12% no change)
				23.5% oropharynx	5.9% ADK	0–1 in 29%;		
				17.6% oral cavity	10.8%	2 in 23.5%		
				11.8 paranasal sinus	Spindle cell carcinoma; 5.9% adenoidcystic carcinoma; 5.9% melanoma; 5.9% acinic cell carcinoma	3 in 47.1%		
				11.8% lip				
				5.9 nasal cavity				
				5.9% salivary gland				
Toya (21)	Retrospective	34	81 (54–92)	56% oral cavity	82% SCC	ECOG:	No	PS 0–1 in 13 pts; 2–3 in 21 pts; No G3 toxicity TR 85%; OR 94%; Symptom relief 77%; Median 0S 5.7 months Median PFS 4.4 months
				15% nasal cavity and paranasal sinuses	18% Others	0 in 15%		
				12% hypopharynx		1 in 24%		
				6% skin		2 in 32%		
				6% major salivary gland		3 in 29%		
				3% thyroid				
				3% neck disease with an unknown primary				
Lok (34)	Retrospective study	75	72 (23–97)	Salivary gland 11%	SCC 65%	KPS <70, 27% >70, 73%	64%	G3 in 5% 65% palliative response Median OS 5.67 months
				Thyroid carcinoma 21%				
				Sarcoma 3%				
				Other 19%				
Kil (26)	Retrospective case control	1	85	Parotid gland	SCC	ECOG 3	No	Improvement of PS (1–2) No toxicity Complete palliative response
Corry (35)	Prospective study	30	73 (52–88)	43% oral cavity, 27% oropharynx, 20% hypopharynx, 3% larynx, 7% unknown	SCC	WHO 0 in 7%, 1 in 27%, 2 in 43%, 3 in 23%	No	67% WHO improved or stabilized G3–4 toxicities in 2 pts OR (objective response) 53% Median OS 5.7 months Median PFS 3.1 months Improved QoL in 44%
Pearson (31)	Retrospective study	15	69 (48–88)	Oropharynx, larynx oral cavity, hypopharynx unknown	SCC	WHO 2–3 in 93%	Unspecificated	Median time to death 4 months Median QoL at last follow-up was good 80% Pain control achieved in 58%
Benhmida (22)	Retrospective	75	80 (74–84)	32% oropharynx; 28% oral cavity; 14.7% hypofarynx; 10.7% larynx; 14.7% others	94.7% SCC	ECOG:	No	G3 toxicity in 5 pts 0S 19.3 months in 95% PFS (pain-free survival) 11.5 months in 95%
					4% ADK	0 in 5.3%		
					1.3% Others	1 in 20.7%		
						2 in 57.3%		
						3 in 5.3%		
						Unspecified in 1.3%		
Bledsoe (27)	Retrospective study	65	71 (42–101)	40% oropharynx	92% SCC	KPS	4 pz concurrent CT	No G4–5 radio toxicity Total TR 91% (50% CR, 41% PR, 9% stable or progressive disease) LFS (median locoregional failure-free survival) 25.7 month OS 8.9 months
				14% hypopharynx	3% ADK	>90 in 17%		
				12% larynx	5% Other	70–80 in 57%		
				9% oral cavity		<60 in 14%		
				8% parotid gland				
				6% nasopharynx				
				3% nasal cavity or paranasal sinus				
				8% Other				
Kancherla (30)	Retrospective study	33	11 < 70 22 > 70	27.3% oral cavity	SCC	WHO >2 in 58%	No	TR: (39% CR, 33% PR) Median OS 9 months PFS at 1 year 35% PFS at 2 years 25%
				24, 2% hypopharynx				
				24.2% larynx				
				18.2% oropharynx				
				6.1% nasopharynx				
Garcia-Anaya (23)	Retrospective study	106	74 (44–93)	1,9% nasopharynx; 17% oropharynx; 7.5% salivary glands; 7.5% hypopharynx; 17% oral cavity; 32.1% larynx; 12.3% skin; 2.8% unknown primary with nodal metastases; 1.9% other	SCC	ECOG	Neoadjuvant (NAD) in 26 pts	PS had a deterioration in 8 pts; No G3 toxicity Median OS 7 months; Median PFS 4.63 months; Complete palliative response for 19.8%; Partial palliative response 59.4%
						0–1 in 32.1%		
						2–3 in 67.9%		
Laursen (36)	Retrospective study	77	73 (47–96)	21% oral cavity	SCC 94%	WHO:	1 pt NAD CT	Median OS 5.4 months Complete loco regional response 31% Partial loco regional response 14%
				1% nasopharynx	Others 6%	0–1 in 29%	4 pts after RT	
				30% oropharynx		2 in 25%		
				12% hypopharynx		3–4 in 15%		
				18% larynx		Missing in 8%		
				8% sinonasal				
				5% salivary glands				
				5% unknown				
VanBeek, (28)	Retrospective study	81	70 (39–92)	26% oral cavity	SCC 89%	Median KPS 70	6 pts NAD CT	Palliative effect 63% TR complete 32%; partial 40%; mixed 8% OR 88% Median OS 7.2 months
				12% salivary glands	Non-SCC 11%	(30–100)		
				16% oropharynx				
				26% hypopharynx				
				3% nasal cavity				
				17% larynx				
Porceddu (33)	Prospective phase II study	37	68 (43–87)	27% oral cavity	SCC	WHO	No	Primary TR (CR + PR) 74% Nodal response (CR+PR) 63% OOR (overall objective response) 80% QoL (62% overall improvement, 14% no change, 24% deterioration) PS (19% improved, 38% never improved, 41% deteriorated, 3% not recorded)
				32% oropharynx		0 in 19%		
				3% glottic larynx		1 in 51%		
				5% supraglottic larynx		2 in 24%		
				16% hypopharynx		3 in 5%		
				16% unknown primary with nodal metastases				
De Felice (24)	Prospective trial	6	77,5 (71 – 82)	50% Oral cavity, 50% oropharynx	SCC	WHO >2	No (but cetuximab 400 mg/m^2^ 1 week before RT; 250 mg/m^2^ weekly during HFRT)	Median PFS 2 months; Median OS 2,5 months. with 3 pz PR and 3 progression 5 death during follow up
Bonomo (25)	Retrospective cohort trial	36	77.5 (65–91)	50% oral cavity	SCC	ECOG	No	No G4–G5 toxicity, but G3 in 36% OS at 6 months 58% OS at 1 year 50% LRC (loco regional control) at 6-months 42% LRC at 1-year 28% PFS at 6 months 36% PFS at 1 year 20%
				16.6% oropharynx		1 in 22.2%		
				16.6% larynx		2 in 52.8%		
				16.6% other		3 in 25%		
Ermiş (29)	Retrospective study	132	65 (33–89)	Glottic carcinoma	SCC	ECOG:	No	RC (regional control) rate at 5 year 85.6% LC rate a 95.4%, CSS 95.7% OS 78.8%
						0–1 in 88%		
						2 in 3%		
						3 in 2%		
						Unknown in 8%		
Al-Magmani (32)	Prospective study	158	68.5 (41–95)	31% oropharynx	SCC	WHO 30 pz ≤2	16 pz induction CT with 2–5 courses of cisplatin and 5-fluorouracil	PS Improvement in 47% OR rate 73% (45% CR and 28% PR, 6% stable disease, and 21% progressed) Median ST (survival time) 17 months LRC at 1 year 62% LRC at 3 years 32% DFS at 1 year 32% DFS at 3 year x14% OS at 1 year 40% OS at 3 years 17%
				23% oral cavity				
				23% hypopharynx				
				20% larynx				
				2% nasopharynx				
				1% nasal cavity and PNS				

PS, performance status; ECOG, Eastern Cooperative Oncology Group; CT, chemotherapy; SCC, squamous cell carcinoma; ADK, adenocarcinoma; OR, overall response; CR, complete response; PR, partial response; KPS, Karnofsky Performance Score; OS, overall survival; TR, tumor response; PFS, progression free survival; QoL, quality of life; LFS, median locoregional failure-free survival; OOR, overall objective response; LRC, locoregional control; RC, regional control; CSS, cause-specific survival.

Details of RT treatment and doses are listed in **Table 2**.

**Table 2:** Details RT treatment and BEDs dose.

	Dose/fraction	No. of fractions	Dose tot	BED10	BED12	EQD2 (10)	EQD2 (12)	No. of courses	Weeks interval between courses	Notes
Ferro (20)	5	4	20	30.0	28.3	25.0	23.6	2	4	Twice daily in repeated cycles
Toya (21)	3.7	4	14.8	20.3	19.4	16.9	16.1	3	3–4	Twice daily in repeated cycles
Lock (34)	3.7	12	44.4	60.8	58.1	50.7	48.4	3	2	Twice daily in repeated cycles
Kil (26)	3.7	4	14.8	20.3	19.4	16.9	16.1	3	4	Twice daily in repeated cycles
Corry (35)	3.7	4	14.8	20.3	19.4	16.9	16.1	3	4	Twice daily in repeated cycles
Pearson (31)	3.7	4	14.8	20.3	19.4	16.9	16.1	3	2	Twice daily in repeated cycles
Benhmida (22)	3	10	30	39.0	37.5	32.5	31.3	2	2–4	Daily in repeated cycles
Bledsoe (27)	3	10	30	39.0	37.5	32.5	31.3	2	3–5	Daily in repeated cycles
	3	12	36	46.8	45.0	39.0	37.5	2	3–5	Daily in repeated cycles
Kancherla (30)	4	5	20	28.0	26.7	23.3	22.2	2	2	Daily in repeated cycles
Garcia Anaya (23)	6	5	30	48.0	45.0	40.0	37.5	1		Twice weekly
	6	6	36	57.6	54.0	48.0	45.0	1		Twice weekly
Laursen (36)	4	13	52	72.8	69.3	60.7	57.8	1		Twice weekly
	4	14	56	78.4	74.7	65.3	62.2	1		Twice weekly
	4	5	20	28.0	26.7	23.3	22.2	1		Twice weekly
van Beek (28)	4	12	48	67.2	64.0	56.0	53.3	1		3–4 times a week
Porceddu (33)	6	6	36	57.6	54.0	48.0	45.0	1		Twice Weekly
	6	5	30	48.0	45.0	40.0	37.5	1		Twice Weekly
	6	4	24	38.4	36.0	32.0	30.0	1		Twice Weekly
	6	3	18	28.8	27.0	24.0	22.5	1		Twice Weekly
De Felice (24)	3	20	60	78.0	75.0	65.0	62.5	1		Consecutive Days
Bonomo (25)	2.5	16	40	50.0	48.3	41.7	40.3	1		Consecutive Days
	2.5	8	20	25.0	24.2	20.8	20.1	1		Consecutive Days
	2.5	13	32.5	40.6	39.3	33.9	32.7	1		Consecutive Days
Ermis (29)	2.75	20	55	70.1	67.6	58.4	56.3	1		Consecutive Days
Al-Magmani (32)	3.125	16	50	65.6	63.0	54.7	52.5	1		Consecutive Days

BED, biologically effective dose; EQD2, equivalent dose in 2 Gy fractions.

### Twice Daily in Repeated Cycles

Ferro et al. studied a cohort of 17 patients with histologically confirmed H&N cancers, aged ≥80 years (range, 80–97 years), and presenting an Eastern Cooperative Oncology Group (ECOG) performance status of ≤3 with an expected survival of >3 months. The first group received a total dose of 20 Gy in 2 consecutive days with a twice-daily fractionation (5 Gy per fraction) and an interval of 8 h between the two.

In the case of absence of toxicity of grade 3 (G3) or higher according to the European Organization for Research and Treatment of Cancer and Radiation Therapy Oncology Group scales (EORTC-RTOG), patients received a second RT cycle after a month, up to a total dose of 40 Gy.

Treatment volume did not include lymph nodes, and the technique used for the treatment was 3D conformal in half of the patients, while IMRT was used in the remaining patients, in which large irradiation fields were expected (20).

A total of nine patients with a median age of 83 years were treated with one cycle, while eight patients with median age of 88 years were treated with two RT courses.

No G3 toxicity was reported in both groups, and follow-up was performed every 2 months for all the patients.

The overall 3-month symptoms-free survival (SFS) was 83.3% and 87.5% for the first and second cohort, respectively.

Five patients had complete response (CR) and 10 partial response (PR), while no change was observed in the remaining 2. Thirteen patients reported pain prior to RT, and their symptoms were partially or completely solved in eight of them. This experience suggests therefore that short course accelerated RT is a safe and overall well-tolerated palliative therapy option for H&N elderly patients.

In this frame, several studies proposed a quad shot, a palliative fractionation scheme.

Toya et al. reviewed a group of 34 patients with H&N cancer treated with the Radiation Therapy Oncology Group (RTOG) 8502 “quad shot” regimen. The most common histological type was squamous cell carcinoma (SCC) (82%), and the median age was 81 years (range, 35–92).

The proposed RT treatment included 3.7 Gy fractions, delivered twice daily with an interval of 6 h between the two fractions, for two consecutive days. This cycle was repeated every 3–4 weeks for three courses, using volumetric modulated arc therapy (VMAT) technique. No concurrent systemic therapy was foreseen.

Eighteen percent of the patients completed one course; 15%, two courses; and 68% completed all the three prescribed courses. Tumor response (TR) was observed in 85% of the patients, and 77% of them had pain relief after the treatment. Overall response (OR) was 94%, and median overall survival (OS) was 5.7 months. Median progression-free survival (PFS) was 4.4 months, and better PFS outcomes were observed only at the completion of both courses. No G3 toxicity was registered (21).

Moreover, Lok et al. investigated the efficacy of the RTOG 8502 quad shot regimen on a group of 75 patients with median age of 76 years. Out of them, 28 patients completed the three courses of RTOG 8502 regimen, and 65% of them had a symptomatic response. Median overall survival was 5.67 months, and G3 toxicity was registered in 5% of the cases (34).

An interesting case report of an old man, affected by SCC of the left parotid gland and presenting multiple comorbidities, was described by Kil et al. The patient was 85 years old and considered unsuitable for CT or surgery and therefore addressed to IMRT quad shot, with 14 Gy in four fractions, twice daily with a 6-h gap, for 2 consecutive days. This course was repeated every 4 weeks for three times. At the follow-up evaluation done 12 months after RT, facial pain appeared to be relieved, and the patient showed no signs of late toxicity with an overall good quality of life. The performance status improved from ECOG 3 to ECOG 1–2. IMRT quad shot appeared, therefore, to be a good and safe palliative treatment for elderly patients presenting comorbidities (26).

Corry et al. evaluated a group of 30 patients affected by SCC, mainly localized in the oral cavity (43%), with a median age of 73 years and presenting a WHO performance status >2 in 66%. Sixteen patients completed all the foreseen radiation courses, and 53% of patients underwent an objective response.

Median overall survival and median progression-free survival were 5.7 and 3.1 months, respectively. Twenty-three patients had disease reduction or stability. Quality of life (QoL) improved in 44% of cases, and WHO performance status remained unchanged or improved in 67% of patients. Only two of them presented G3–G4 toxicity during the follow-up (35).

Another series of 15 patients affected by H&N SCC was studied by Pearson et al. The predominant primary site was in this case oropharynx (33%); median age was 69 years, and 93% of the patients had performance status 2–3. The treatment used for these patients consisted of three phases of hypofractionated split course regimen.

Every phase had a dose of 14.8 Gy in four daily fractions with a dose of 3.7 Gy per fraction and an interval of 2 weeks between the courses. The target volume included the primary tumor and the involved lymph nodes. Eleven patients completed the treatment, two died before completing the first two phases. In 58% of the patients pain relief was observed, while quality of life improved in six patients. These observations support split course hypofractionation as a valid alternative for palliation with favorable toxicity profile (31).

### Daily in Repeated Cycles

Benhimida et al. retrospectively analyzed the efficacy of split course RT regimen. Seventy-five patients with a median age of 80 years were treated with intensity modulated radiotherapy (IMRT). Two courses of 30 Gy in 10 fractions with an interval of 2/4 weeks between them. Median follow-up was 10.6 months and OS values at 12 and 24 months of 60.4% and 41.5%, respectively. Median PFS was 11.5 months (47.7% at 12 months and 41% at 24 months). Ninety-six percent of the patients completed the regimen, and 16% needed hospitalization during the treatment. Grade 3 skin toxicity was observed in only one patient. Three patients showed G3 late toxicity and one osteoradionecrosis of mandible occurred 6 months after the RT (22).

A retrospective study by Bledsoe et al. described an original regimen of split course accelerated HFRT for 65 patients with H&N cancer, unsuitable for standard treatments. The most common histology was SCC, and the major sites involved were oropharynx (40%), hypopharynx (14%), and larynx (12%). The median age was 71 years. The treatment consisted of 60–72 Gy in 20–24 fractions, divided in two courses with 3–5 weeks of pause. Sixty-five patients with a median age of 71 years (42–101) were treated with this schedule, and 58 of them completed both courses.

Four patients underwent concurrent CT, while 15 patients (23%) underwent surgery prior to RT. Total TR was 91%. Median locoregional failure-free survival was 25.7 months and OS was 8.9 months. Fifty percent of patients had a CR, 41%, a PR, and 9% had stable or progressive disease. No G4-G5 toxicity were observed (27).

Kancherla et al. reported about 33 patients with H&N SCC, unsuitable for curative treatment and underwent split course HFRT. Twenty-two patients were over 70 years old. The regimen included a first step of 20 Gy in 5 fractions over 1 week, followed by a 2-week gap and a second treatment of 20 Gy in 5 fractions over 1 week.

The performance status was 2–3 (WHO) in 58% patients, and the median age was 76. The most common tumor primary sites were oral cavity, hypopharynx, and larynx. All patients completed the foreseen treatment cycles. Thirty-nine 39% of patients presented a complete tumour response while 33% partial.

The PFS rates at 1 and 2 years were 35 and 25%, respectively. Median OS was 9 months.

Only eight cases of G3 toxicity were observed in the entire cohort. In 79% of the cases, the symptoms improved, while only 6% of patients had symptom deterioration, and 15% did not undergo any symptomatic change (30).

### Alternate Days

The study of Garcia-Anaya et al. reported the outcomes of 106 patients with H&N cancer, poor ECOG, or advanced age treated with HFRT schedule. The median age was 74 years.

Of the patients, 53.8% received 30 Gy in five fractions of 6 Gy, twice weekly. The remaining 46.2% were treated with 36 Gy in six fractions.

Twenty-six patients underwent CT prior to radiation treatment. Nodal disease was included in treatment volume in 41 patients. Of the patients, 10.4% did not complete the prescribed regimen due to ECOG deterioration, exitus, voluntary cessation, or hospitalization.

Of the patients, 19.8% had complete palliative response, and the remaining 59.4% had a PR. Median OS was 7 months, and PFS was 4.63 months. Like other studies, this paper reported major palliative response for doses higher than 30 Gy and ECOG 0–1 PS. Interestingly, no G3 skin toxicity was reported, and no deaths associated with radiation treatment have been observed (23).

In their retrospective study, Laursen et al. reported a series of 77 patients with H&N cancer undergoing palliative HFRT. The primary tumor site was oropharynx in 29.9%, oral cavity in 20.8%, and larynx in 11.7%. The most common histology was SCC (93.5%). Age ranged from 47 to 96 years, with a median of 73.

A treatment with 53–56 Gy in 13–14 fractions twice weekly was administered in these patients, and 75% of them completed the treatment.

One patient received CT before RT, and two received adjuvant CT. Locoregional control (LRC) was 45%, and PR was reached in 14% of cases. The median OS was 5.4 months, 1 year survival was 31%, and 2 years survival was 18% (36).

Van beek et al. evaluated an alternative HFRT treatment for 81 patients affected by H&N cancer.

The median age was 70 years. The proposed total dose was 48 Gy divided in 12 fractions of 4 Gy each, three to four times per week.

Six of them underwent palliative CT before radiation treatment, and 11 patients underwent surgery. Palliative effect occurred in 63% of them. CR and PR were 32% and 40%, respectively. OR rate was 88%, and OS was 7.2 months. Toxicity related to radiation treatment occurred more often in patients treated with 2D/3D technique than IMRT, suggesting that it as possible technical gold standard (28).

Porceddu et al. studied 35 patients with incurable H&N SCC. The predominant primary site was oropharynx (32%), and the median age was 68 years (43–97). The patients underwent 30 Gy in five fractions in 2 weeks with an additional boost of 6 Gy: 19 patients received 36 Gy; 12 of them, 30 Gy; 3, 24 Gy; and 1, 18 Gy. OR was 80%, and 37% of patients showed G3 toxicity. Thirteen patients had an overall QoL improvement, no changes were observed in three, and five deteriorated; 67% of them had an improvement in overall pain, and WHO performance status improved in 19% of cases, while it deteriorated in 41% (33).

### Consecutive Days

De Felice et al. reported the objective response in vulnerable elderly patients affected by H&N cancer, who underwent HFRT combined with cetuximab. Six patients with locally advanced squamous H&N carcinoma were included in this study. Median age was 77.5 years.

The treatment plan consisted of a total dose of 60 Gy (3 Gy/fraction) associated with concurrent cetuximab (400 mg/m^2^ 1 week before HFRT, followed by 250 mg/m^2^ during the treatment). All patients completed the foreseen RT schedule, 3% of patients completed the treatment with cetuximab, while one patient received only the first dose.

PR was observed in three patients, while the remaining three underwent progression disease. No CR was observed. Median objective PR (OPR) duration was 4.5 months.

There were four cases of severe toxic effects, and five deaths were recorded during the follow-up.

Median PFS rate was 2 months, and median OS was 2.5 months (24).

Bonomo et al. studied a group of 36 patients with locally advanced squamous H&N carcinoma, unsuitable for chemoradiotherapy or high-dose RT. The major primary site was oral cavity (50%), and the median age was 77.5 years.

The performance status was ECOG 2–3 in 77.8%. Three-dimensional conformal RT was used in 69.4% of cases, while VMAT was preferred in 30.6%. The radiation schedule included 2.5 Gy/fraction for 16 fractions, with a total dose of 40 Gy to primary tumor volume and involved nodes.

Thirty-three patients completed the treatment; two patients interrupted at 20 Gy and one case at 32.5 Gy. Four patients had CR and 18 PR, with an OR of 66.6%. The LRT rate at 6 months was 42%, and 28% at 1 year. PFS at 6 months and 1 year were 36% and 20%, respectively. No G4–G5 toxicity was observed. Median OS was 12 months. This retrospective study confirmed the overall clinical benefit and low toxicity profile of HFRT even in frail and elderly patients (25).

In the study of Ermis et al., a group of 130 patients with T1/T2 N0 SCC of the glottis and a median age of 65 years (33–89) underwent HFRT with a dose of 55 Gy in 20 fractions at 2.75 Gy per fraction over 4 weeks. Five years LC was 85.6%, and ultimate LC was 97.3%. Five years regional control, CSS, and OS rates were 95.4%, 95.7%, and 78.8%, respectively. Of the patients, 99% had complete clinical response. During the 10 years follow-up, 15 patients had recurrence, 36 of them died, and 17% of them had second cancers. Grade 3 toxicities were registered in 22 cases (29).

Lastly, Al-Magmani et al. analyzed the outcomes of a HFRT schedule on a group of 158 patients affected by H&N SCC, unsuitable for curative treatment. The most frequent involved site was oropharynx (31%), and the median age was 68.5 years.

The treatment planning (Christie schemes) consisted of 3.1 Gy in 16 fractions for a total dose of 40 Gy. Of the patients, 45% had CR, 28% had PR, 6% had a stable disease, and 21% had progression. Median survival time was 17 months, and 40% of patients survived 1 year after RT. At 1 year, LRC rate was 62%, DFS was 32%, and OS was 40%. At 3 years, LRC was 32%, DFS 14%, and OS 17%. Grade 3 toxicity was observed in 45% of the patients and severe late toxicity in 4.5% only (32).

## Discussion

This review analyzes the use of HFRT in the elderly patients, aiming to assess the safety and feasibility of hypofractionated treatment regimens with radical aim in this patient setting. Grewal et al. recently reviewed studies that proposed palliative treatments in incurable patients and demonstrated that hypofractionated courses were promising for patients with poor performance status and prognosis (13).

While Grewal’s aim was to evaluate treatment schemes with palliative intent, this review targets the hypofractionated treatment schemes with radical intent that may successfully be implemented in elderly patients.

Most patients in the studies indeed did not receive CT due to age and comorbidities. Elderly patients, despite the intrinsic age-related frailty, may often be suitable for high-dose radical treatments. The studies analyzed in this systematic review are mostly with palliative intent, low EQD2, and total BED palliative purposes. Nevertheless, these treatments succeeded in achieving prolonged disease control.

Among these, the studies that are closest to the topic of our review are the studies by De Felice and Ermis with BED_10_ of 78 Gy and 70.1, respectively.

It can be noted that in the treatment proposed by De Felice et al., four out of six patients presented severe toxicity, while the results of the study by Ermis et al. are more satisfactory in terms of both efficacy and safety, with RTOG G3 skin toxicity occurring in nine (6.8%) patients, even if this latest study had a glottic larynx only as target volume, therefore limiting the generalizability of observed results to the general H&N population. Furthermore, in De Felice’s study, patients underwent concomitant CT and had a significantly older age (77.5 *vs*. 65 years). All the differences do not allow an adequate comparison between these studies, but the scheme used by Ermis seems particularly suitable for the specific topic of our review.

Despite the existing differences, both studies have indeed a radical treatment intent and are relevant for this specific patients subpopulation, conversely to the palliative treatments of Grewal’s review.

The 55 Gy at 2.75 Gy per fraction scheme over 4 weeks has also been proposed for other types of H&N cancer in several studies (14, 37–42). In these studies, the mean age is <60 years, and specific references on elderly patients are not discussed in details However, these studies showed an overall good tolerability and could enhance elderly patients’ compliance by reducing the number of needed accesses to the radiotherapy facility. The HFRT schedule is currently being tested in the ongoing International Atomic Energy Agency (IAEA) multicentric trial of hypo- *vs* normo-fractionated accelerated RT in non-nasopharyngeal HNSCC (HYPNO study), registered at clinical trials.gov (NCT0765503). The results of this study may be useful in consolidating this treatment schedule, although patients with major comorbidities are excluded from recruitment, thereby limiting its value in terms of particularly frail categories. A prospective randomized trial in elderly patients would be therefore particularly useful but difficult to perform for enrollment reasons and timing.

In conclusion, we highlighted new valid HFRT schedules with palliative purposes that should be added to the algorithm proposed in the study by Grewal et al. (13).

In elderly patients fit for radical treatments, a 55 Gy in 20 fractions treatment can be proposed as a valid alternative to the standard fractionated regimens, even if there still is a multitude of hypofractionated regimens, ranging from single fraction, quad shot, and 1-, 2-, 3-, 4-, and 5-week schedules that all may be considered appropriate.

The results of our review still do not allow us to establish the most convenient treatment scheme in this patient setting, with a strong dependence on many factors including patient’s performance status, disease extent, ability to come for radiation treatment, concurrent or future systemic therapy plans, and overall life expectancy. To imply that a single regimen is the preferred alternative would not be accurate, given the nuanced decision making that is required when selecting a treatment regimen for a given frail patient.

From our results, we recommend therefore to follow the algorithm proposed by Grewal to support the decision making for palliative treatments. On the other hand, we suggest the scheme of 55 Gy in 20 fractions as the most promising one when considering a treatment with radical intent, even if further studies are needed to consolidate this indication.

## Data Availability Statement

The original contributions presented in the study are included in the article/supplementary material. Further inquiries can be directed to the corresponding author.

## Author Contributions

Study concepts: AP. Study design: AP. Data acquisition: VV and SM. Quality control of data and algorithms: AD, GP, and SM. Data analysis and interpretation: AP, VV, and SM. Statistical analysis: AP, SM, and VV. Manuscript preparation: VV, AP, and LB. Manuscript editing: AP and LB. Manuscript review: LB, TA, AD, and CF. All authors contributed to the article and approved the submitted version.

## Conflict of Interest

The authors declare that the research was conducted in the absence of any commercial or financial relationships that could be construed as a potential conflict of interest.

## Publisher’s Note

All claims expressed in this article are solely those of the authors and do not necessarily represent those of their affiliated organizations, or those of the publisher, the editors and the reviewers. Any product that may be evaluated in this article, or claim that may be made by its manufacturer, is not guaranteed or endorsed by the publisher.
